# The serum C1q-to-CRP ratio performs well for diagnosing periprosthetic joint infection in revision arthroplasty

**DOI:** 10.3389/fcimb.2026.1831300

**Published:** 2026-07-09

**Authors:** Xiaodong Jia, Shuhang Dong, Jinli Chen, Xia Zhao, Yingze Zhang, Kuishuai Xu

**Affiliations:** 1 Knee Preservation Center, The Affiliated Hospital of Qingdao University, Qingdao, Shandong, China; 2 Department of Sports Medicine, The Affiliated Hospital of Qingdao University, Qingdao, Shandong, China; 3 Department of Orthopedics, The Third Affiliated Hospital of Hebei Medical University, Shijiazhuang, Hebei, China

**Keywords:** biomarker ratio, complement C1q, CRP, diagnosis, periprosthetic joint infection

## Abstract

**Background:**

Periprosthetic joint infection (PJI) is a severe complication after total joint arthroplasty (TJA). Conventional biomarkers like C-reactive protein (CRP) and erythrocyte sedimentation rate (ESR) often show limited accuracy in complex cases. Complement C1q is a critical initiator of the classical complement pathway, but its diagnostic value in PJI has not been investigated.

**Methods:**

We retrospectively analyzed 168 patients undergoing revision arthroplasty (71 PJI and 97 aseptic loosening [AL]). Preoperative serum levels of C1q, CRP, ESR, and fibrinogen (FIB) were measured. Diagnostic models including the C1q-to-CRP ratio (CCR), ESR-to-C1q ratio (ECR), and FIB-to-C1q ratio (FCR) were calculated. ROC curve analysis and DeLong’s test were used to assess diagnostic performance. Decision curve analysis (DCA) was conducted to assess the clinical net benefit of the CCR model. An exploratory stratified analysis was further performed in the diabetic subgroup (n=48).

**Results:**

Serum C1q levels were significantly higher in the PJI group compared to the AL group (236.00 vs. 180.00 mg/L, P < 0.001). C1q positively correlated with CRP (rho = 0.62), ESR (rho = 0.33), and FIB (rho = 0.31). The CCR model demonstrated the highest diagnostic accuracy with an AUC of 0.949, which was significantly superior to CRP alone (AUC = 0.931, P = 0.022). At a cut-off of 0.03, CCR yielded a sensitivity of 95.77% and a negative predictive value of 96.20%. In the diabetic subgroup, CCR remained effective with an AUC of 0.928.

**Conclusion:**

Serum C1q is a reliable novel biomarker for PJI. The CCR model demonstrates promising research prospects and can serve as a reliable tool for clinical decision-making in the diagnosis and treatment of PJI, including for diabetic patients. DCA showed that CCR provided superior net clinical benefit across most threshold probabilities in both the overall and diabetic cohorts, supporting its potential clinical utility in PJI diagnosis.

## 1 Introduction

Total joint arthroplasty (TJA) is a cornerstone of modern orthopedics, effectively restoring function and alleviating pain in the rapidly growing population with end-stage osteoarthritis (Kapadia et al., 2016; Patel, 2023). However, periprosthetic joint infection (PJI), despite its relatively low incidence (0.5%–2%), remains a devastating complication. It not only compromises surgical outcomes but also imposes an immense clinical and socioeconomic burden (Whitehouse et al., 2016; Gundtoft et al., 2017; Koh et al., 2017). Consequently, the early, rapid, and precise diagnosis of PJI is paramount to mitigating these adverse impacts.

Over the past decades, research into PJI diagnostics has expanded significantly, yielding diversemodalities such as novel serological and synovial biomarkers, PET-CT, polymerase chain reaction,mass spectrometry, and next-generation sequencing (Esteban andGomez-Barrena, 2021; Feng et al., 2025; Zhang et al., 2025b; Giatroudakis et al., 2026; Zhu et al.,2026). Nevertheless, establishing a timely and accurate diagnosis remains a formidablechallenge due to the lack of a universal “gold standard.” Among current methods, peripheral blood testing remains the clinical mainstay for hospitalized patients owing to its accessibility, cost-effectiveness, and rapid turnaround. While consensus statements widely recommend C-reactive protein (CRP) and erythrocyte sedimentation rate (ESR) for screening, their diagnostic performance in complex PJI cases has proven limited (Elmenawi et al., 2026). In addition to traditional inflammatory markers, fibrinogen (FIB), D-dimer, serum amyloid A, calprotectin, and alpha-defensin have all demonstrated potential diagnostic value (Xu et al., 2021; Moldovan, 2024; Paul et al., 2025; Zhang et al., 2025b; Giatroudakis et al., 2026). Furthermore, to improve diagnostic accuracy, several ratio-based models derived from inflammatory biomarkers, such as the CRP-to-albumin ratio and albumin-to-globulin ratio, have been proposed. Nevertheless, the existing biomarkers still exhibit limitations in terms of sensitivity, specificity, and diagnostic stability across different clinical settings. These limitations are particularly evident in patients with chronic PJI, low-virulence infections, or multiple comorbidities. Therefore, the identification of novel biomarkers that reflect distinct pathophysiological mechanisms and provide improved diagnostic performance remains an important research priority in the field of PJI.

Complement C1q, a key initiator of the classical complement pathway, has emerged as a high-fidelity biomarker in various inflammatory and neoplastic diseases. In the context of infection, C1q is a reliable prognostic indicator for sepsis mortality and a potential therapeutic target (Trzeciak et al., 2022). Furthermore, C1q has been linked to neuroinflammation—specifically associated with cerebrospinal fluid sTREM2 and the mediation of amyloid-β/tau pathology—and is significantly elevated in the peripheral blood of patients with active tuberculosis compared to healthy or latent controls (Cai et al., 2014; Guo et al., 2025). In oncology, C1q-like 4 expression serves as a potential marker for breast cancer malignancy (Zhang et al., 2025a), while elevated serum C1q levels have also been observed in major depressive disorders (Yao and Li, 2020). Compared with complement components involved in downstream stages of the complement cascade, C1q is more closely associated with the initial phase of infection-related immune responses and may therefore provide a more sensitive indication of pathogen-induced immune activation.

Despite its established role in systemic inflammation, the diagnostic utility of complement C1q in PJI remains completely unexplored. Therefore, this study performed a retrospective analysis of serological data to evaluate the diagnostic efficacy of C1q, either independently or in combination with CRP, ESR, and FIB. We hypothesize that a multi-marker panel integrating C1q will yield superior diagnostic precision compared to traditional standalone markers (CRP, ESR and FIB), which may provide a more robust tool for the clinical diagnosis and management of PJI.

## 2 Methods

### 2.1 Patient selection and study design

This retrospective study was conducted to evaluate the diagnostic efficacy of serum biomarkers in identifying PJI. Patients undergoing revision arthroplasty at our institution between January 2015 and December 2024 were screened. A total of 168 patients undergoing revision arthroplasty were enrolled and divided into two cohorts: the PJI group and the aseptic loosening (AL) group. The diagnostic criteria for PJI are based on the standards established by the Musculoskeletal Infection Society (MSIS) (Parvizi et al., 2014), while AL was defined as mechanical failure without evidence of infection. Furthermore, a specific diabetic subgroup was analyzed to assess biomarker performance in the presence of metabolic comorbidities. This study was conducted in strict accordance with the ethical principles of the Declaration of Helsinki for medical research involving humans and was approved by the Ethics Committee of The Affiliated Hospital of Qingdao University (QYFY WZLL 42077).

### 2.2 Inclusion and exclusion criteria

**Figure 1:**
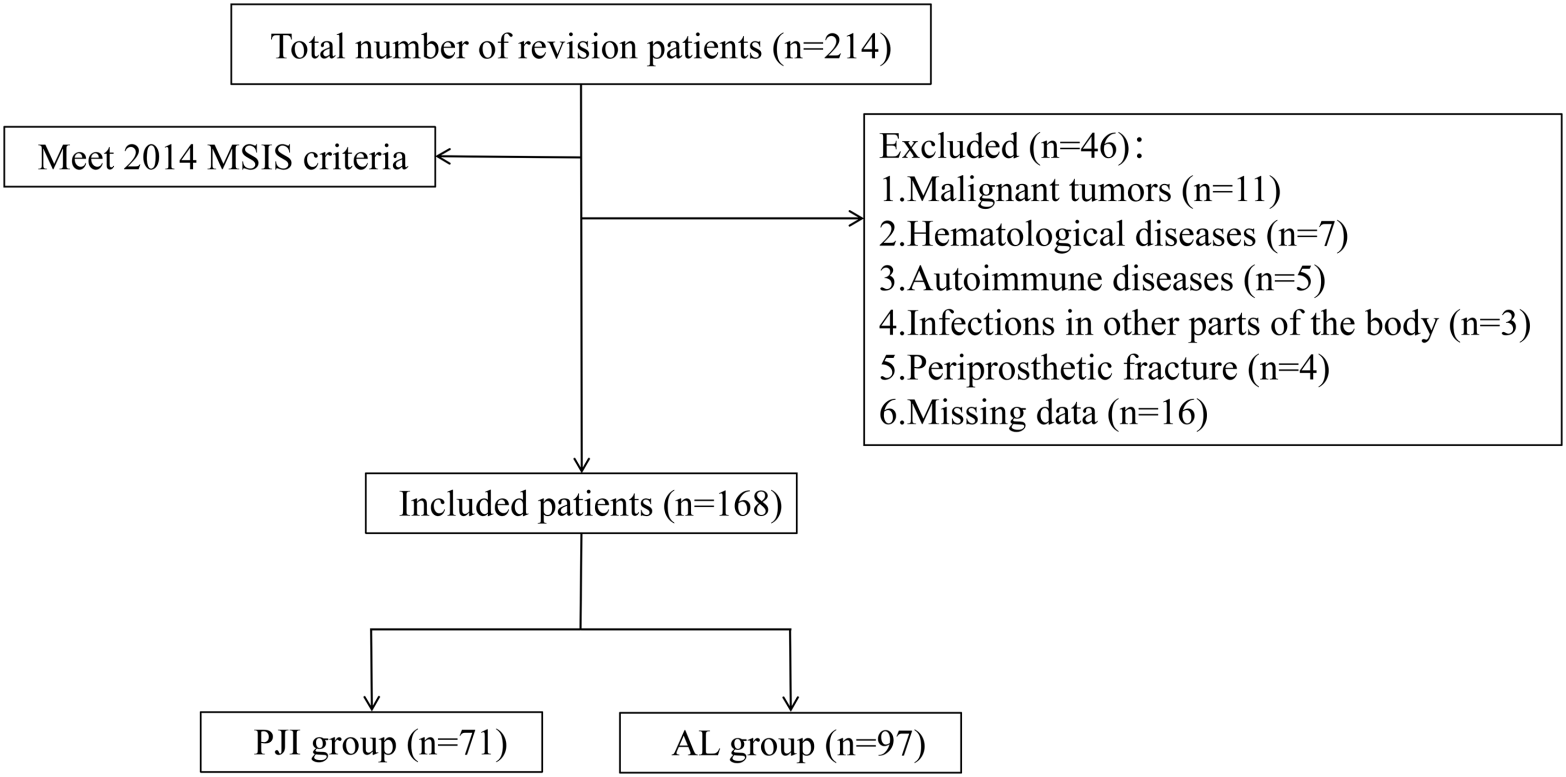
Flowchart of patient inclusion.

The flowchart of the inclusion and exclusion process is illustrated in **Figure 1**. The specific criteria were as follows: Inclusion criteria: (1) a confirmed diagnosis of PJI or AL and receipt of corresponding treatment; and (2) availability of complete clinical data. Exclusion criteria: (1) presence of malignant tumors; (2) presence of hematological diseases; (3) presence of autoimmune diseases; (4) presence of infections in other parts of the body; (5) presence of periprosthetic fracture; (6) missing clinical data.

### 2.3 Data collection, baseline characteristics and laboratory measurements and biomarker ratios

Comprehensive demographic and clinical data were abstracted from electronic medical records. Key variables included age, sex, surgical site, and comorbidities such as obesity, diabetes, hypertension, and cardiovascular disease. To ensure the reliability of subsequent biomarker evaluations, the demographic homogeneity and clinical comparability between the PJI and AL cohorts were rigorously verified. Preoperative peripheral blood samples were collected from all patients. Standard laboratory assays were used to quantify traditional inflammatory markers, including CRP, ESR and FIB, alongside complement C1q levels. To explore potential synergistic effects, three ratio-based models were calculated: the C1q-to-CRP ratio (CCR), the ESR-to-C1q ratio (ECR), and the FIB-to-C1q ratio (FCR). To minimize potential bias, the laboratory personnel performing the biomarker measurements were blinded to the patients’ clinical diagnoses and group assignments.

### 2.4 Statistical analysis

Statistical analyses were performed using R software (version 4.5.2; R Foundation for Statistical Computing, Vienna, Austria). Continuous variables were expressed as medians with interquartile ranges (IQRs) and compared using the Mann-Whitney U test. Categorical variables were compared using the Chi-square or Fisher’s exact test. The diagnostic performance of each marker was quantified by Receiver Operating Characteristic (ROC) curve analysis, calculating the Area Under the Curve (AUC), sensitivity, and negative predictive value (NPV). A *post hoc* ROC power analysis indicated that the study achieved sufficient statistical power (>0.99). The relationship between serum C1q and conventional markers (CRP, ESR, and FIB) was evaluated using Spearman’s rank correlation analysis. In addition to the whole-population analysis, a focused investigation was conducted within the diabetic subgroup to determine if the inflammatory response and diagnostic accuracy of these markers were maintained in patients with impaired glucose metabolism. DeLong’s test was employed to compare the diagnostic accuracy between individual markers and the ratio-based models. DCA was performed to evaluate the clinical utility and net benefit of the CCR model. DCA was conducted by quantifying the net benefit across a range of threshold probabilities and comparing the CCR model with the “treat-all” and “treat-none” strategies. The net benefit was calculated based on the relative harms of false-positive and false-negative classifications. DCA was performed for both the overall cohort and the diabetic subgroup to assess the potential clinical applicability of the CCR model in different patient populations. A P-value < 0.05 was considered statistically significant.

## 3 Results

### 3.1 Comparative analysis of patient demographics, baseline characteristics, and serum biomarkers

**Figure 2:**
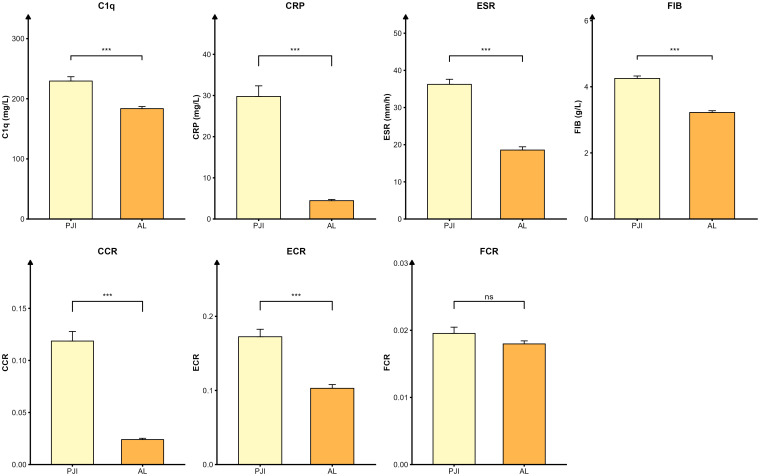
Comparison of levels of different markers between the PJI group and the AL group: CCR,CRP/C1q; ECR, ESR/C1q; FCR, FIB/C1q. ns, not significant (P ≥ 0.05); ***P < 0.001.

A total of 168 patients were enrolled in this study, including 71 in the PJI cohort and 97 in the AL cohort. As shown in **Table 1**, no statistically significant differences were observed between the two groups regarding age, gender distribution, surgical site and key comorbidities—including obesity, diabetes, hypertension, and cardiovascular disease (all P > 0.05, **Table 1**; **Figure 2**). These results underscore the demographic homogeneity of the cohorts, providing a rigorous baseline for subsequent biomarker evaluation. Conversely, serum biomarker profiles differed markedly between groups. Compared with the AL group, the PJI group exhibited significantly elevated levels of C1q, CRP, ESR and FIB, with all comparisons reaching high statistical significance (all *P* < 0.001). Specifically, the median serum C1q level in PJI patients was 236.00 (182.50–283.50) mg/L, significantly higher than the 180.00 (160.00–202.00) mg/L in the AL group. Similar significant elevations were noted for CRP (31.26 vs. 3.54 mg/L), ESR (41.00 vs. 16.00 mm/h), and FIB (4.36 vs. 3.22 g/L). Furthermore, the CCR and ECR were significantly higher in the PJI group (*P* < 0.001), while no significant difference was observed in the FCR between groups (*P* = 0.765).

**Table 1:** Basic characteristics of the PJI group and the AL group.

Variables	PJI (n=71)	AL (n=97)	p
Age (years)	67.00 (63.00, 68.00)	65.00 (62.00, 68.00)	0.163
Gender			0.203
Male	23 (32.39%)	42 (43.30%)	
Female	48 (67.61%)	55 (56.70%)	
Location			0.297
Knee	26 (36.62%)	27 (27.84%)	
Hip	45 (63.38%)	70 (72.16%)	
Obesity			0.154
Yes	19 (26.76%)	16 (16.49%)	
No	52 (73.24%)	81 (83.51%)	
Diabetes			0.071
Yes	26 (36.62%)	22 (22.68%)	
No	45 (63.38%)	75 (77.32%)	
Hypertension			0.348
Yes	27 (38.03%)	29 (29.90%)	
No	44 (61.97%)	68 (70.10%)	
CVD			1.000
Yes	20 (28.17%)	27 (27.84%)	
No	51 (71.83%)	70 (72.16%)	
C1Q (mg/L)	236.00 (182.50, 283.50)	180.00 (160.00, 202.00)	<0.001
CRP (mg/L)	31.26 (7.01, 42.53)	3.54 (2.88, 4.64)	<0.001
ESR (mm/h)	41.00 (25.00, 46.00)	16.00 (12.00, 21.00)	<0.001
FIB (g/L)	4.36 (3.83, 4.78)	3.22 (2.89, 3.56)	<0.001
CCR	0.12 (0.05, 0.18)	0.02 (0.02, 0.03)	<0.001
ECR	0.15 (0.11, 0.20)	0.09 (0.07, 0.12)	<0.001
FCR	0.02 (0.02, 0.02)	0.02 (0.02, 0.02)	0.765

### 3.2 Correlation analysis between serum C1q and conventional inflammatory markers

**Figure 3:**
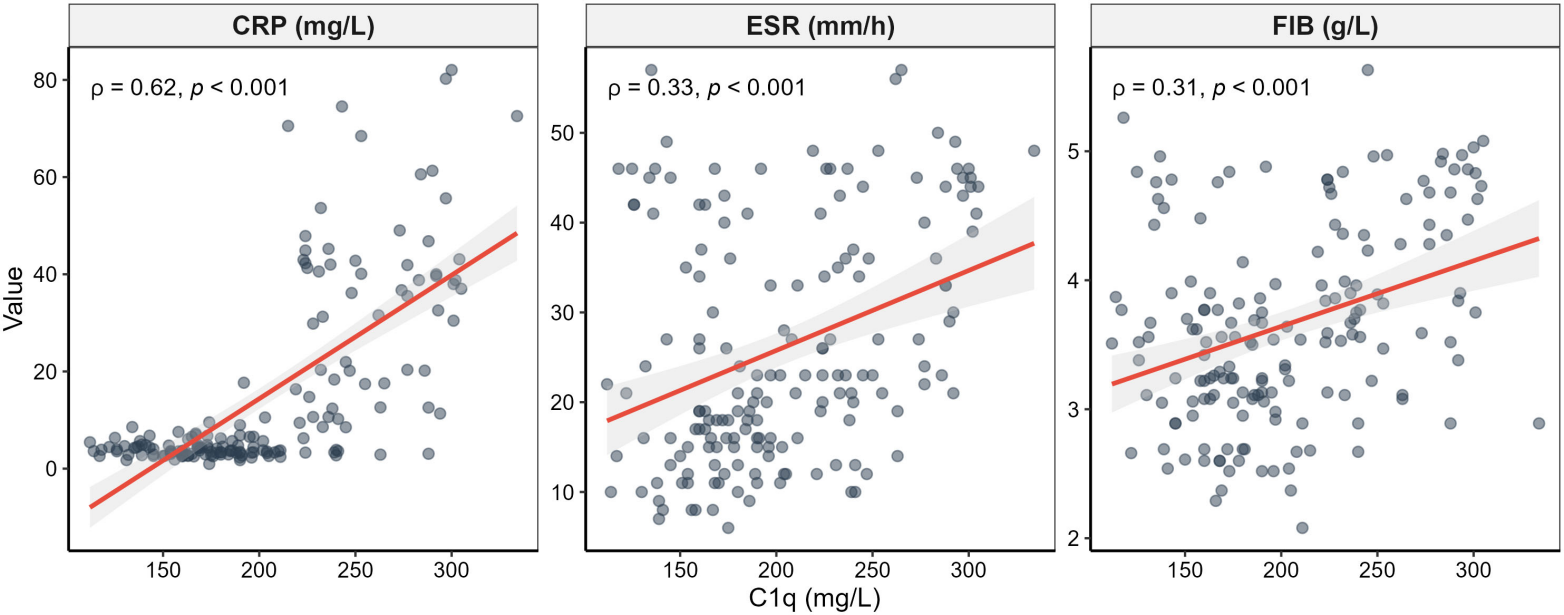
Correlation analysis between serum C1q levels and levels of CRP, ESR, and FIB.

Spearman correlation analysis was employed to assess the relationship between serum C1q levels and traditional inflammatory markers (CRP, ESR and FIB). Serum C1q demonstrated a significant positive correlation with all evaluated markers (all *P* < 0.001, **Figure 3**). Among these, C1q showed the strongest correlation with CRP (*ρ* = 0.62, *P* < 0.001). Additionally, serum C1q exhibited moderate positive correlations with ESR (*ρ* = 0.33, *P* < 0.001) and FIB (*ρ* = 0.31, *P* < 0.001).

### 3.3 Evaluation of diagnostic efficacy for all biomarkers in the overall population

**Figure 4:**
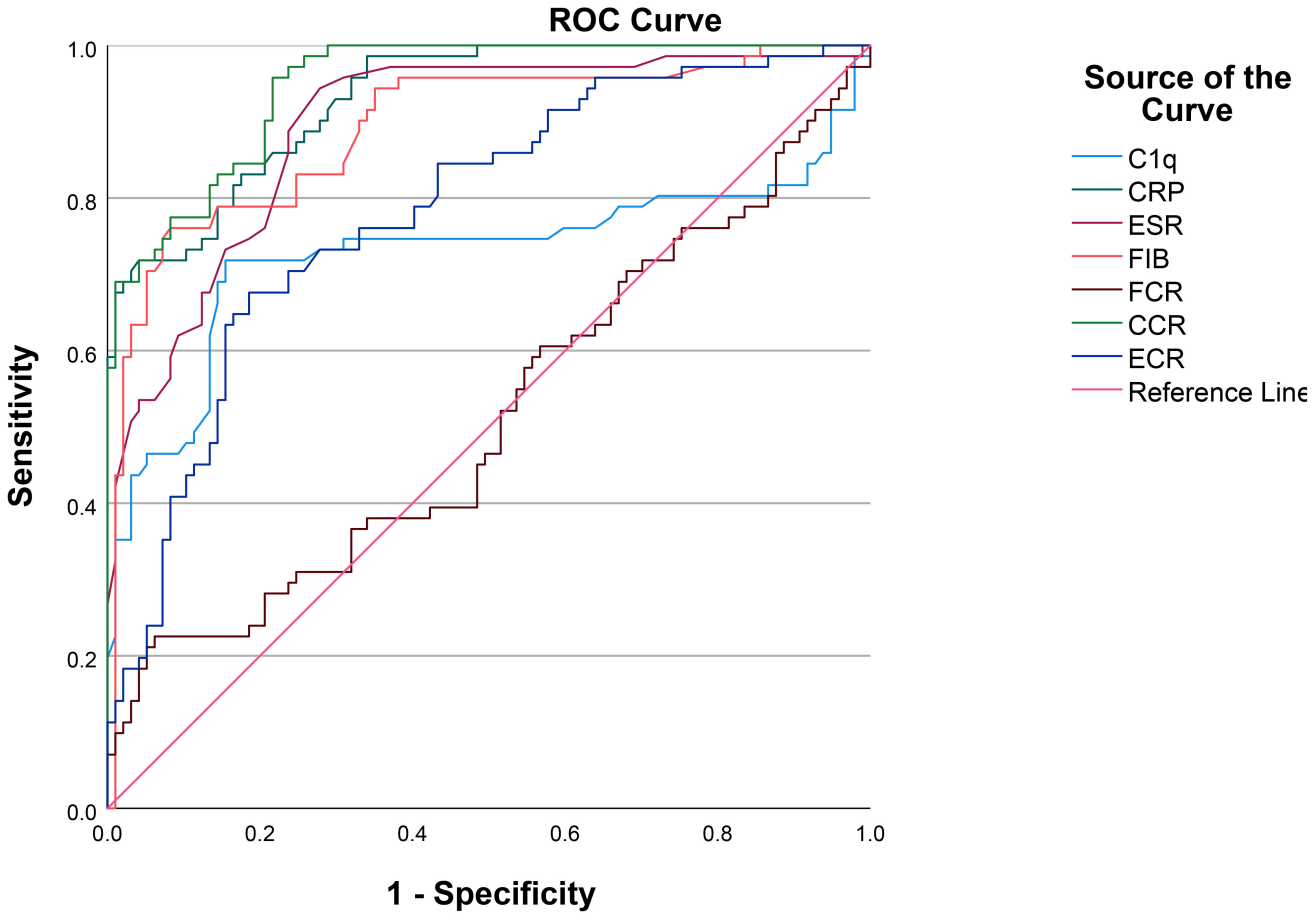
The ROC curves of C1q, CRP, ESR, FIB, CCR, ECR, FCR. CCR, CRP/C1q; ECR, ESR/C1q; FCR, FIB/C1q.

**Figure 5:**
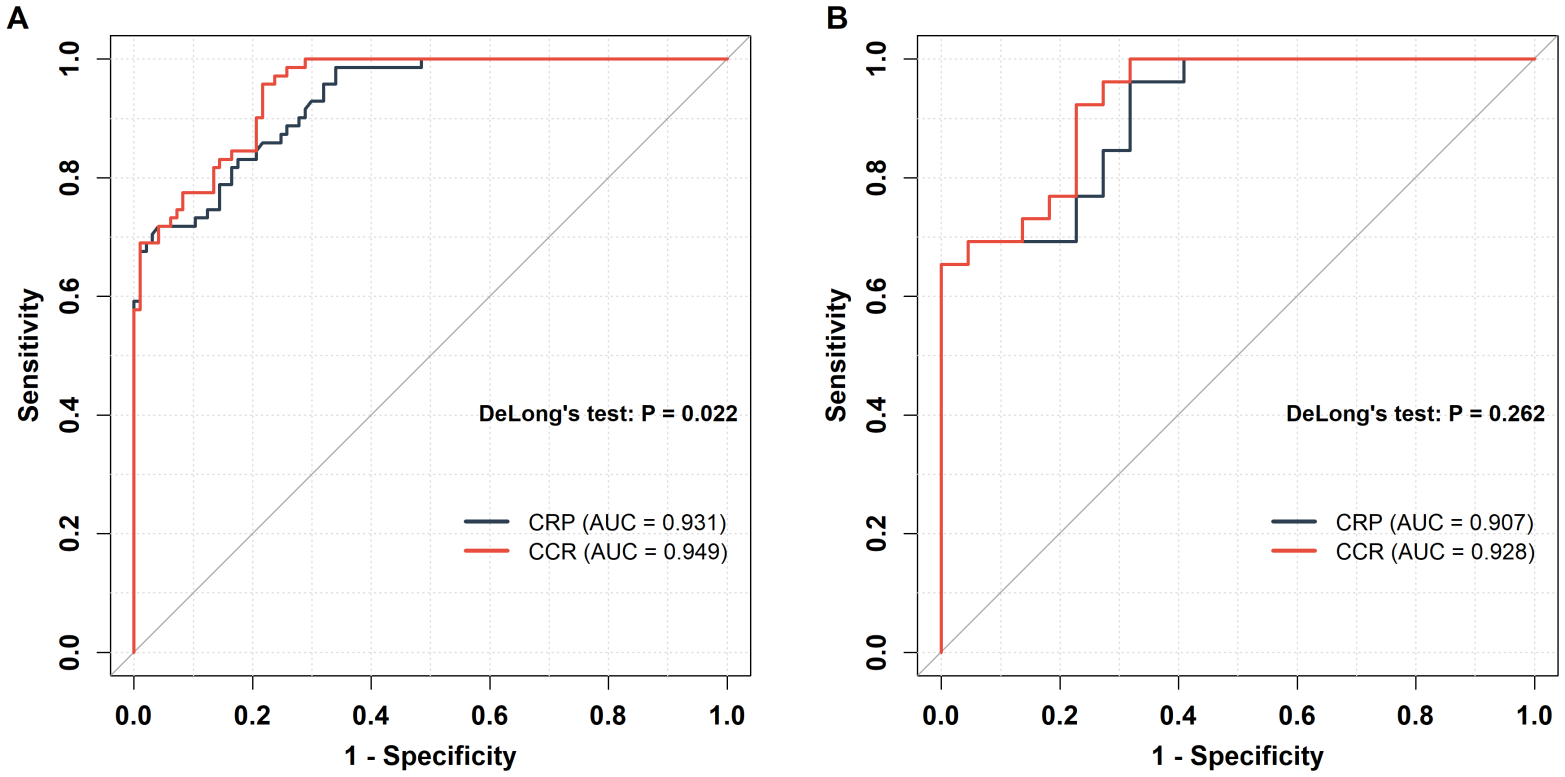
Comparison of diagnostic accuracy using DeLong’s test. **(A)** Total cohort; **(B)** Diabetic subgroup. AUC, Area Under the Curve; CCR, CRP/C1q.

The diagnostic performance of each marker was quantified using ROC analysis (**Table 2**; **Figure 4**). Among the independent markers, CRP achieved the highest accuracy (AUC = 0.931, 95% *CI*: 0.896–0.966), with a positive predictive value (PPV) of 92.73% and an NPV of 82.30%, while serum C1q yielded an AUC of 0.728 (95% *CI*: 0.638–0.818), with a PPV of 77.27% and an NPV of 80.39%. Notably, integrating C1q and CRP into a ratio-based model significantly improved diagnostic precision; the CCR emerged as the most robust tool with an AUC of 0.949 (95% *CI*: 0.921–0.977). DeLong’s test confirmed that the diagnostic efficacy of CCR was significantly superior to that of CRP alone (*P* = 0.022, **Figure 5**). At an optimal cut-off value of 0.03,the CCR achieved a sensitivity of 95.77%, a PPV of 76.40%, and an NPV of 96.20%, outperforming all individual serum markers.

**Table 2:** ROC curve analysis of C1q for PJI.

Factors	AUC (95% CI)	Cut-off	Youden index	Sensitivity (%)	Specificity (%)	PPV (%)	NPV (%)
C1q	0.728 (0.638-0.818)	213.00	0.56	71.83	84.54	77.27	80.39
CRP	0.931 (0.896-0.966)	10.58	0.68	71.83	95.88	92.73	82.30
ESR	0.893 (0.844-0.942)	20.50	0.67	94.37	72.16	71.28	94.59
FIB	0.892 (0.840-0.944)	3.79	0.68	76.06	91.75	87.10	83.96
CCR	0.949 (0.921-0.977)	0.03	0.74	95.77	78.35	76.40	96.20
ECR	0.783 (0.713-0.853)	0.12	0.49	67.61	81.44	72.73	77.45
FCR	0.486 (0.395-0.578)	0.02	0.09	60.56	48.45	46.24	62.67

### 3.4 Comparison of demographics and biomarkers in the diabetic subgroup

**Figure 6:**
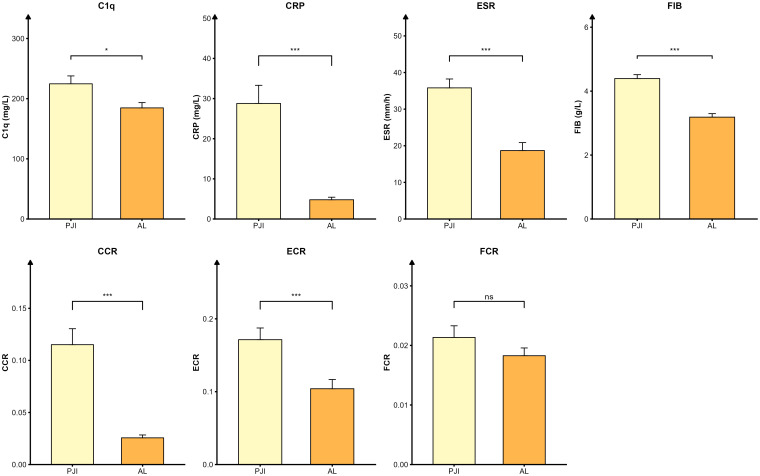
Among the diabetes subgroups, the levels of different markers between the PJI group and the AL group: CCR,CRP/C1q; ECR, ESR/C1q; FCR, FIB/C1q. ns, not significant (P ≥ 0.05); *P < 0.05; ***P < 0.001.

Within the diabetes subgroup, a total of 48 patients were included, comprising 26 in the PJI group and 22 in the AL group (**Table 3**; **Figure 6**). There were no significant differences in age, sex, surgical site and key comorbidities—including obesity, hypertension, and cardiovascular disease between the two cohorts. These results verify the comparability of clinical baselines within this subgroup. In diabetic patients, biomarker levels in the PJI group remained significantly higher than in the AL group. Specifically, C1q (224.50 vs. 176.50, *P* = 0.038), CRP (25.39 vs. 3.58, *P* < 0.001), ESR (40.00 vs. 16.50, *P* < 0.001) and FIB (4.67 vs. 3.11, *P* < 0.001) were all markedly elevated. Similarly, CCR and ECR were significantly higher in the PJI cohort (all *P* < 0.001), while FCR showed no statistical difference (*P* = 0.390).

**Table 3:** Baseline demographic and clinical characteristics of the PJI and AL groups within the diabetes patient population.

Variables	PJI (n=26)	AL (n=22)	P value
Age (years)	63.00 (62.00, 67.00)	65.50 (62.00, 68.00)	0.692
Gender			0.942
Male	12 (46.15%)	9 (40.91%)	
Female	14 (53.85%)	13 (59.09%)	
Location			0.559
Knee	9 (34.62%)	5 (22.73%)	
Hip	17 (65.38%)	17 (77.27%)	
Obesity			0.953
Yes	6 (23.08%)	4 (18.18%)	
No	20 (76.92%)	18 (81.82%)	
Hypertension			0.357
Yes	14 (53.85%)	8 (36.36%)	
No	12 (46.15%)	14 (63.64%)	
CVD			1.000
Yes	10 (38.46%)	9 (40.91%)	
No	16 (61.54%)	13 (59.09%)	
C1q (mg/L)	224.50 (155.25, 292.25)	176.50 (160.00, 190.00)	0.038
CRP (mg/L)	25.39 (6.38, 42.89)	3.58 (2.80, 5.45)	<0.001
ESR (mm/h)	40.00 (23.75, 46.00)	16.50 (10.50, 22.50)	<0.001
FIB (g/L)	4.67 (3.90, 4.86)	3.11 (2.74, 3.48)	<0.001
FCR	0.02 (0.01, 0.03)	0.02 (0.01, 0.02)	0.390
CCR	0.10 (0.04, 0.19)	0.02 (0.02, 0.03)	<0.001
ECR	0.15 (0.12, 0.19)	0.09 (0.06, 0.12)	<0.001

### 3.5 Diagnostic performance in the diabetic subgroup

**Figure 7:**
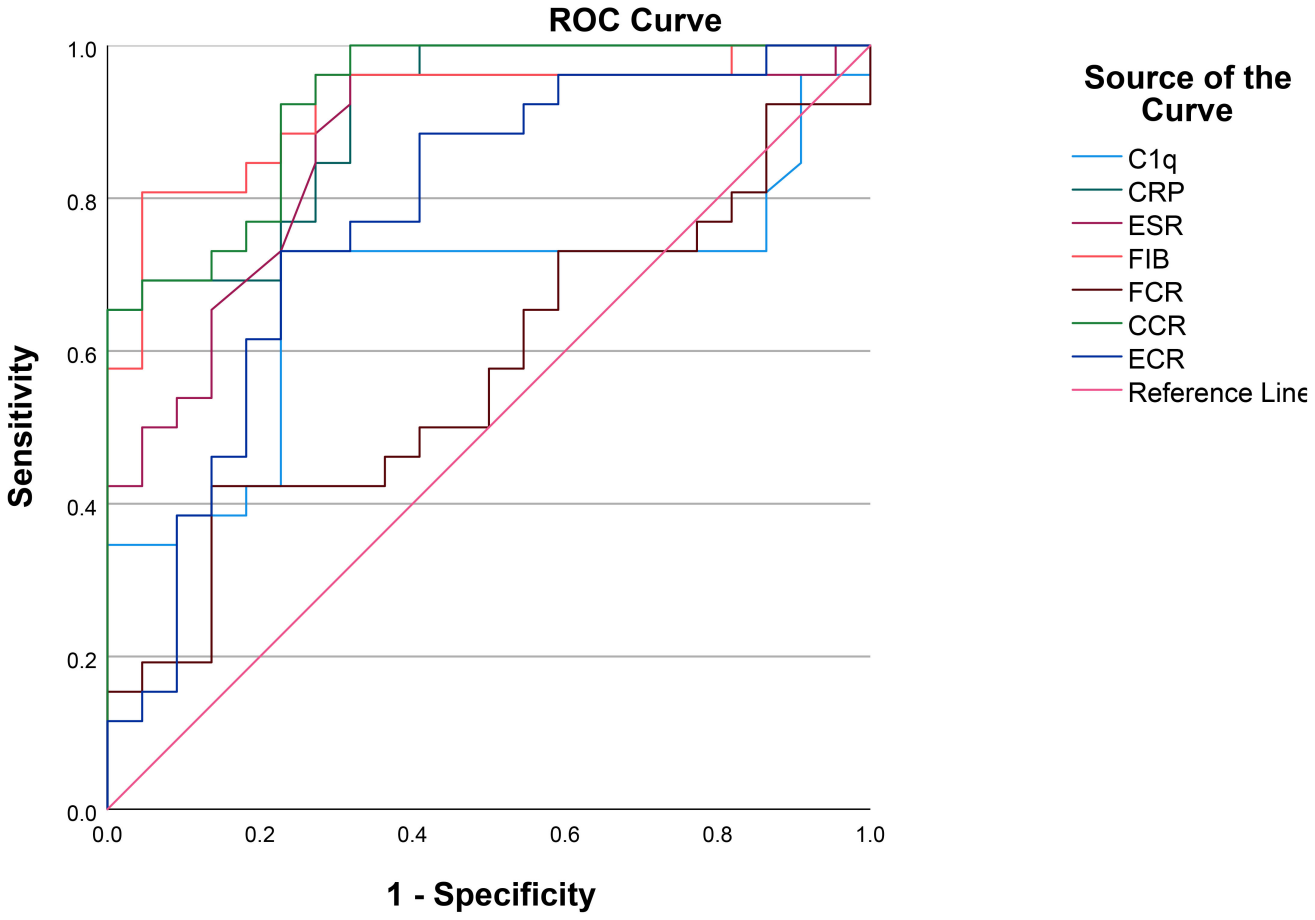
The ROC curves of C1q, CRP, ESR, FIB, CCR, ECR and FCR in the diabetes subgroups. CCR, CRP/C1q; ECR, ESR/C1q; FCR, FIB/C1q.

ROC analysis further quantified marker performance in the diabetic population (**Table 4**; **Figure 7**). Among independent markers, FIB (AUC = 0.921, 95% *CI*: 0.843–1.000, PPV = 95.45%, NPV = 80.77%) and CRP (AUC = 0.907, 95% *CI*: 0.829–0.986, PPV = 100.00%, NPV = 70.97%) demonstrated excellent accuracy, while C1q yielded an AUC of 0.676 (95% *CI*: 0.514–0.838, PPV = 79.17%, NPV = 70.83%). Notably, CCR maintained exceptional diagnostic efficacy in this subgroup with an AUC of 0.928 (95% *CI*: 0.861–0.996). At a cut-off of 0.03, CCR showed a sensitivity of 92.31%, a PPV of 82.76% and an NPV of 89.47%. However, DeLong’s test indicated that the difference in efficacy between CCR and CRP did not reach statistical significance in this subgroup (*P* = 0.262, **Figure 5**). Therefore, although CCR demonstrated a numerically higher AUC and NPV than CRP, its potential incremental diagnostic value in diabetic patients should be interpreted with caution. Further studies with larger sample sizes are required to validate its diagnostic value. Consistent with the overall cohort, FCR lacked clinical utility in diabetic patients (AUC = 0.573, PPV = 78.57%, NPV = 55.88%).

**Table 4:** ROC curve analysis of C1q for PJI within the diabetes patient population.

Factors	AUC (95% CI)	Cut-off	Youden index	Sensitivity (%)	Specificity (%)	PPV (%)	NPV (%)
C1q	0.676 (0.514-0.838)	191.00	0.50	73.08	77.27	79.17	70.83
CRP	0.907 (0.829-0.986)	14.47	0.65	65.38	100.00	100.00	70.97
ESR	0.864 (0.757-0.970)	19.50	0.64	96.15	68.18	78.12	93.75
FIB	0.921 (0.843-1.000)	3.88	0.76	80.77	95.45	95.45	80.77
CCR	0.928 (0.861-0.996)	0.03	0.70	92.31	77.27	82.76	89.47
ECR	0.776 (0.639-0.914)	0.13	0.50	73.08	77.27	79.17	70.83
FCR	0.573 (0.408-0.739)	0.02	0.29	42.31	86.36	78.57	55.88

### 3.6 Evaluation of clinical utility using decision curve analysis

DCA demonstrated that the CCR model provided a higher net clinical benefit across a wide range of threshold probabilities compared with both the “treat-all” and “treat-none” strategies in the overall cohort. Similarly, in the diabetic subgroup, the CCR model also showed a favorable net benefit over most clinically relevant threshold ranges. These findings suggest that CCR may offer potential clinical value in supporting decision-making for the diagnosis of periprosthetic joint infection, particularly when used within an appropriate probability threshold range (**Supplementary Figure 1**).

## 4 Discussion

PJI remains one of the most formidable complications following TJA, and its accurate diagnosis is fundamental to determining the optimal revision strategy. Although the International Consensus Meeting has established standardized criteria, conventional markers such as CRP and ESR often exhibit suboptimal sensitivity and specificity when encountering low-virulence or chronic infections, frequently leading to diagnostic uncertainty (Palan et al., 2019; Elmenawi et al., 2026). In this study, we introduced serum C1q—a central component of the complement system—and developed the first ratio-based diagnostic model, the CCR, to provide a more robust immunological assessment. Our results demonstrate that serum C1q levels are significantly elevated in patients with PJI compared to those with AL, a finding supported by profound pathophysiological mechanisms. The complement system serves as the innate immune system’s primary line of defense, recognizing and clearing pathogens through the classical, alternative, and lectin pathways. C1q acts as the initiating trigger for the classical pathway (Struglics et al., 2016). During the progression of PJI, microbial biofilms on the prosthetic surface continuously induce the host to produce specific antibodies. C1q subsequently recognizes and binds to these antigen-antibody complexes, triggering the complement cascade. This biofilm-mediated immune activation remains persistent and pronounced in PJI patients. In contrast, inflammation in AL is primarily driven by foreign-body reactions to mechanical wear debris, which typically does not involve high-intensity classical complement activation (Zimmerli et al., 1982; Zimmerli and Sendi, 2011). Consequently, the stepwise elevation of C1q levels effectively reflects the intense immunological struggle between the host immune system and the pathogens residing on the prosthetic surface, providing a scientific basis for its use as a differential diagnostic marker.

The most critical breakthrough of this study is the demonstration of the diagnostic superiorityof the CCR. Single biomarkers are often vulnerable to fluctuations in individual basal metabolicrates or non-specific systemic diseases, leading to significant baseline noise. By calculating theratio of C1q (representing immune activation intensity) to CRP (representing systemic inflammatory breadth), we successfully attenuated background noise and amplified the infection-specific signal. DeLong’s test confirmed that the AUC for CCR (0.949) was significantly superior to that of CRP alone (P = 0.022). Several emerging biomarkers have demonstrated promising diagnostic value for PJI. A recent meta-analysis reported that synovial calprotectin achieved an AUC of 0.94, while serum amyloid A yielded an AUC of 0.96 with a sensitivity of 85% and specificity of 96%. Similarly, alpha-defensin demonstrated a pooled sensitivity of 87.8% and specificity of 97.9%. In the present study, CCR achieved an AUC of 0.949 and a sensitivity of 95.77%, suggesting that its diagnostic performance is comparable to that of several currently recognized biomarkers (Xu et al., 2021; Moldovan, 2024; Paul et al., 2025; Zhang et al., 2025b; Giatroudakis et al., 2026).

Correlation analysis revealed a significant positive relationship between C1q and the conventional marker CRP, a phenomenon attributable to the molecular coupling within their respective pathological mechanisms. CRP is an acute-phase reactant synthesized by the liver. Its level increases significantly during inflammation, infection, and tissue injury, making it a classic inflammatory marker (Marnell et al., 2005; Pieri et al., 2014). Studies have suggested that the ratio of CRP to other biomarkers exhibits superior diagnostic efficacy for PJI compared with CRP alone (Luger et al., 2024). However, other studies have demonstrated that its sensitivity and specificity are not significantly better than those of CRP itself (Elmenawi et al., 2026). Existing literature suggests that CRP, upon binding to its ligands, can directly recruit and activate C1q; this synergy ensures that both markers consistently reflect the systemic inflammatory status (Singh et al., 2020). Previous studies have also demonstrated that the concentration of C−reactive protein is positively correlated with complement C1q (Hu et al., 2023). Conversely, C1q demonstrated a lower correlation with ESR and FIB. While ESR is highly susceptible to non-immune interferences such as anemia and hyperglobulinemia (Shahi et al., 2015), C1q—acting as a bridge between innate and adaptive immunity—captures immune activation signals that conventional markers may fail to detect. Furthermore, at an optimal threshold of 0.03, the CCR exhibited an exceptionally high NPV of 96.20%. In clinical practice, a high NPV allows clinicians to rule out PJI with greater confidence when results fall below the threshold, thereby avoiding high-risk, high-cost two-stage revision surgeries and achieving a balance of diagnostic efficiency and patient safety.

Complement C1q has been confirmed to be closely associated with various diabetes-related complications. Studies have shown that C1q levels are significantly elevated in patients with diabetes complicated by acute ischemic stroke and can serve as an independent associated factor for the occurrence of acute ischemic stroke (Hu et al., 2023). Meanwhile, the activation status of C1q is closely related to the occurrence and severity of diabetic nephropathy, suggesting that C1q activation participates in the pathological process of renal injury in diabetic patients (Yang et al., 2019). Analysis of the high-risk diabetic subgroup further validated the robustness of the model. Diabetic patients often exist in a state of immune dysregulation due to chronic hyperglycemia-induced microvascular damage and neutrophil dysfunction; thus, their baseline inflammatory markers are typically higher than the general population (Lontchi-Yimagou et al., 2013). Although the diabetic environment may lead to non-enzymatic glycation of complement components, the CCR maintained a high AUC of 0.928 in this subgroup. While the difference between CCR and CRP did not reach statistical significance in this specific cohort (P = 0.262), this was likely limited by the smaller subgroup sample size. Viewed as a trend, the stability of CCR in the context of metabolic disorders remained superior to single markers, suggesting that for complex cases involving diabetes, the multi-dimensional assessment provided by CCR can effectively reduce the rate of missed diagnoses.

Additionally, our comparative evaluation of the ECR and FCR offered significant academic insights. Although the ECR showed differences between groups, the FCR failed to demonstrate statistical significance. This indicates that simple combinations of various indicators do not inherently yield diagnostic gains; the pathological trajectories of FIB and C1q during infection may be too parallel, rendering the ratio method ineffective. A possible explanation is that both FIB and CRP are positive acute-phase reactants regulated by similar inflammatory pathways and tend to increase concurrently during PJI. Consequently, constructing a ratio between these two biomarkers may attenuate their diagnostic signals, resulting in a lower discriminatory ability of FCR compared with FIB alone. In contrast, the success of the CCR model stems from the nuanced differences in the pathological response timelines and intensities of C1q and CRP, forming a complementary core competency.

## 5 Limitations

While this study is the first to validate the utility of serum C1q and the CCR model alongside traditional and fibrinolytic biomarkers, several limitations must be acknowledged. First, as a single-center retrospective study, it is subject to inherent data biases. Therefore, prospective multicenter validation and independent external validation cohorts are needed to further confirm our findings. Second, the sample size requires expansion to verify the universality of these findings across different ethnic and regional populations. Third, the study did not stratify results by pathogen species (e.g., drug-resistant vs. sensitive strains, or bacteria vs. fungi); different pathogens may vary in their degree of complement consumption and activation. Fourth, future research should prioritize prospective cohorts to facilitate dynamic monitoring of C1q and CCR, exploring the fluctuation curves of CCR at various postoperative time points and its potential in evaluating the efficacy of anti-infective therapy. Finally, comparative studies between local complement levels in synovial fluid and systemic serum levels remain an important future direction.

## 6 Conclusion

In summary, serum complement C1q, particularly when combined with CRP in the CCR model, shows potential as a novel biomarker for the diagnosis of PJI. However, further prospective multicenter studies are required before CCR can be recommended for routine clinical use.

## Data Availability

The raw data supporting the conclusions of this article will be made available by the authors, without undue reservation.

## Supplementary material

Supplementary Figure 1 The decision curve analysis (DCA) was performed in two groups. **(A)** DCA for the overall cohort; **(B)** DCA for the diabetes subpopulation. The prediction model provided superior standardized net benefit compared with the “treat all” and “treat none” strategies in both groups.
