# Supplementary figures and images for: The serum C1q-to-CRP ratio performs well for diagnosing periprosthetic joint infection in revision arthroplasty
Source: Front Cell Infect Microbiol. 2026 Jul 9;16:1831300. doi: 10.3389/fcimb.2026.1831300 (PMC13391870; doi:10.3389/fcimb.2026.1831300)

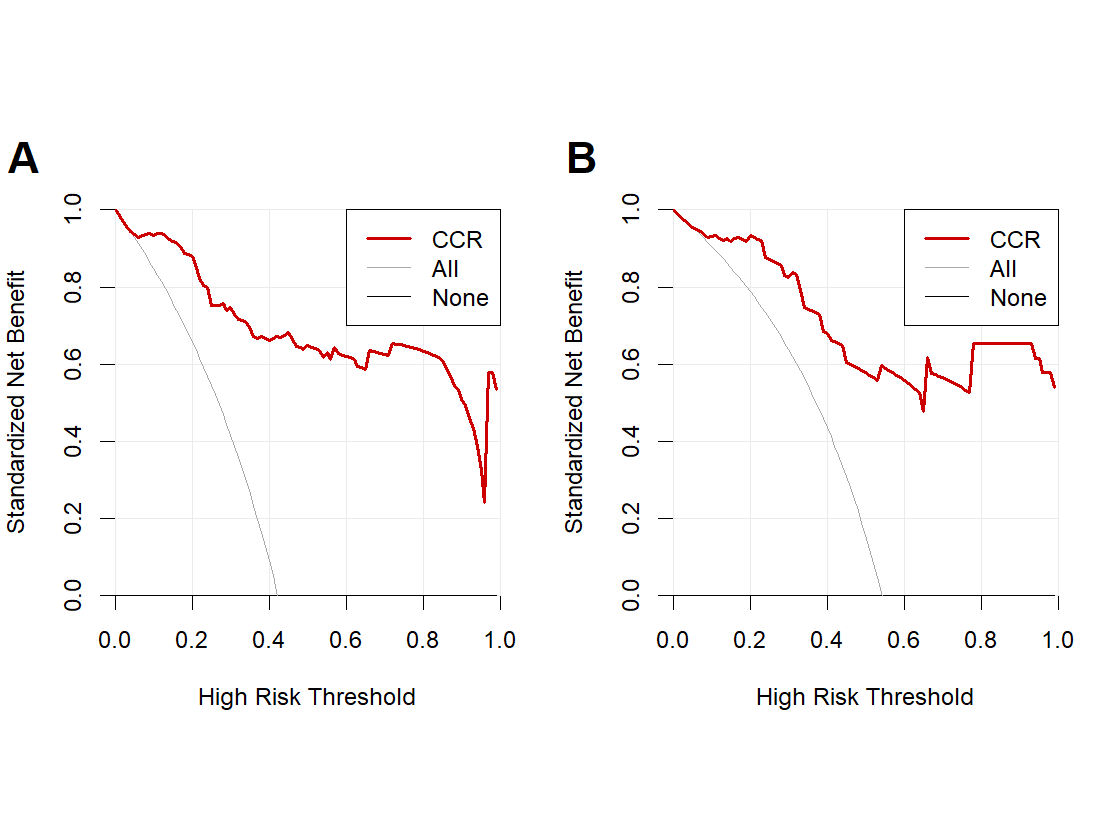

Supplement: Supplementary Figure 1 — The decision curve analysis (DCA) was performed in two groups. (A) DCA for the overall cohort; (B) DCA for the diabetes subpopulation. The prediction model provided superior standardized net benefit compared with the “treat all” and “treat none” strategies in both groups. [file Image1.tiff]
